# High prevalence of Y-box protein-1/p18 fragment in plasma of patients with malignancies of different origin

**DOI:** 10.1186/1471-2407-14-33

**Published:** 2014-01-20

**Authors:** Frank Tacke, Oliver Galm, Nicolas Kanig, Eray Yagmur, Sabine Brandt, Jonathan A Lindquist, Christiane S Eberhardt, Ute Raffetseder, Peter R Mertens

**Affiliations:** 1 Medical Clinic III, University Hospital RWTH-Aachen, Pauwelsstrasse 30, 52074 Aachen, Germany; 2 Medical Clinic IV, University Hospital RWTH-Aachen, Pauwelsstrasse 30, 52074 Aachen, Germany; 3 Department of Nephrology and Hypertension, Diabetes and Endocrinology, Otto-von-Guericke University Magdeburg, Leipziger Str. 44, 39120 Magdeburg, Germany; 4 Medical Care Center, Dr. Stein and colleagues, 41061 Mönchengladbach, Germany; 5 Medical Clinic II, University Hospital RWTH-Aachen, Pauwelsstrasse 30, 52074 Aachen, Germany

**Keywords:** Cold shock proteins, Cancer disease, Serum biomarker, Cancer screening, Prognosis, YB-1

## Abstract

**Background:**

Expression of the cold shock protein Y-box protein 1 (YB-1) is associated with deleterious outcome in various malignant diseases. Our group recently showed that the detection of an 18 kDa YB-1 fragment (YB-1/p18) in human plasma identifies patients with malignant diseases. We now tested the prevalence, clinical, and diagnostic value of YB-1/p18 detection in common tumors.

**Methods:**

A newly established monoclonal YB-1 antibody was used to detect YB-1/p18 by immunoblotting in plasma samples from 151 unselected tumor patients, alongside established tumor markers and various diagnostic measures, during evaluation for a cancerous disease and in follow-up studies after therapeutic interventions.

**Results:**

Circulating YB-1/p18 was detected in 78% of patients having a tumor disease. YB-1/p18 positivity was highly prevalent in all examined malignancies, including lung cancer (32/37; 87%), breast cancer (7/10; 70%), cancer of unknown primary (CUP; 5/5, 100%) or hematological malignancies (42/62; 68%). Positivity for YB-1/p18 was independent of other routine laboratory parameters, tumor stage, or histology. In comparison to 13 established tumor markers (cancer antigens 15–3, 19–9, 72–4, and 125; carcinoembryonic antigen; cytokeratin fragments 21–1; neuron-specific enolase; alpha-fetoprotein; beta-2-microglobulin; squamous cell carcinoma antigen; thymidine kinase; tissue polypeptide antigen; pro-gastrin-releasing peptide), YB-1/p18 detection within serum samples was the most sensitive general parameter identifying malignant disorders. YB-1/p18 concentrations altered during therapeutic interventions, but did not predict prognosis.

**Conclusions:**

Plasma YB-1/p18 detection has a high specific prevalence in malignancies, thereby providing a novel tool for cancer screening independent of the tumor origin.

## Background

Cold shock proteins are evolutionarily conserved, and share a so-called cold shock domain [1,2]. In humans, three members of the protein family have been described, denoted DNA-binding protein A (DbpA) (also called zona occludens 1-associated nucleic acid binding protein (ZONAB) or cold shock domain A (CSDA)), DbpB (Y-box protein-1, YB-1), and DbpC (Contrin). Whereas initial studies dealt with the transcriptional activities of cold shock proteins, i.e. their association with the DNA promoter elements of various target genes, it has become clear that cold shock proteins also associate with mRNA and thereby influence the half-life of mRNA as well as affect pre-mRNA splicing [3]. Transcription rates of proliferation-associated genes are upregulated by YB-1, e.g. DNA-polymerase-α, epidermal growth-factor receptor, platelet-derived growth factor, and matrix metalloproteinase-2 [1,2]. A pivotal role of YB-1 in cancerogenesis has been proposed by several groups, which has been substantiated by its interplay with c-Myc expression in multiple myeloma, as well as p53 function/signaling in malignant melanoma [4,5]. YB-1 facilitates the binding of wild type p53 to DNA motifs, however not of mutated p53, and thereby represses cell death-associated fas gene transcription. The first hint for the participation of cold shock proteins in cancerogenesis and the promotion of metastasis formation has been described in breast cancer disease, as YB-1 expression correlates with cell transformation and confers aggressive tumor growth [6,7]. The overexpression of (mostly nuclear) YB-1 has been associated with poor outcome, e.g. early relapses and aggressive tumor growth, in several tumor entities (summarized in [2]). For instance, nuclear YB-1 expression in tumor tissue from patients with non-small cell lung cancer were associated with disease progression, proliferation markers and prognosis [8-10].

Cold shock proteins may also be actively secreted by both transformed and non-transformed cells following challenge with cytokines (e.g. PDGF-B, TGF-β) or exposure to oxidative stress [11]. YB-1 lacks an N-terminal signal peptide motif and therefore its secretion is regulated similar to that of other leaderless proteins, including interleukin-1β, high mobility group box protein (HMGB1) and macrophage migratory inhibitory factor (MIF). In addition to the full-length YB-1 protein, we have also detected protein fragments in conditioned cell culture medium [11]. In a recent pilot study, we were able to demonstrate that the detection of YB-1/p18 fragment was able to identify patients with malignancies in a well defined cohort of patients with chronic liver diseases awaiting liver transplantation [12]. The band at 18 kDa was identified as the truncated cold-shock domain with peptides corresponding to aa81-137 of the YB-1 protein [12]. In contrast, YB-1/p18 was almost undetectable in human plasma from healthy volunteers, patients with inflammatory diseases, renal, or hepatic failure. We therefore hypothesized that YB-1/p18 detection might represent a novel, yet unrecognized characteristic of patients with malignancies. In order to test this hypothesis, we have conducted the current study in which we tested the prevalence, clinical, and diagnostic value of YB-1/p18 detection in common tumor entities using a novel immunoblotting system.

## Methods

### Patients

The study protocol was approved by the local ethics committee and conducted in accordance with the ethical standards laid down in the 1974 Declaration of Helsinki (ethics committee of the University Hospital Aachen, RWTH-University, Aachen, Germany, reference number EK 107/05). Written informed consent was obtained from each participant. Plasma samples were obtained from consecutive patients with various malignant disorders presenting to the Outpatient Cancer Clinic at the University Hospital Aachen, Germany. Only patients with a histologically confirmed diagnosis of a malignancy were included. Concomitant diseases, routine laboratory tests, tumor staging, and current treatment as well as treatment history were recorded. Blood samples were collected in EDTA plasma separator tubes, and plasma was stored at -80°C. In 42/151 patients, samples were also obtained during follow-up visits (median 3 samples from different time-points). All patients were followed for at least 12 months after inclusion in the study to assess the predictive value of YB-1/p18 and other tumor markers on survival.

### YB-1 immunoblotting

Human plasma (0.5 μl diluted 1:10 with ddH_2_O) was separated on 12.5% SDS-polyacrylamide gels and transferred to nitrocellulose membranes. Following blocking for 1 h with 2.5% milk in Tris-buffered saline the membranes were incubated with primary antibody, monoclonal anti-YB-1 ( [13], Portugal (II 2C-5)/ Biotin 1.3 g/ml Lot 1 A1 biotinylated, 1:1000) overnight at 4°C. After extensive washing with TBST, peroxidase-conjugated Streptavidin (Dianova, 1:10,000) was incubated for 1 h at room temperature. Detection was performed with the ECL system (Amersham).

**Figure 1:**
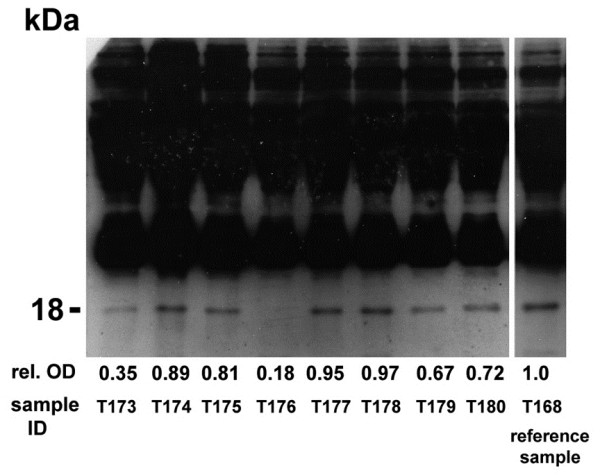
**YB-1/p18 detection in human plasma.** Human plasma was blotted onto a nitrocellulose membrane and YB-1 detected by immunoblotting using a novel monoclonal antibody. Patients with malignant disorders often displayed an additional signal at 18 kDa (“YB-1/p18”), and its intensity was quantified by densitometry. A positive reference sample was run on each blot (T168). Samples with an 18-kD band ≥0.45 were considered to be YB-1/p18 positive.

On each blot, one sample obtained from a patient with metastasized small cell lung cancer that was strongly YB-1/p18 positive was run in parallel as a positive control (Figure 1). The immunoblots were performed and analyzed by a scientist blinded to the origin of the samples. YB-1/p18 signals were quantified by densitometry (NIH imager) and compared to the positive control, which was assigned the optical density of ′1.0′. The relative optical density of the samples was calculated and signals ≥0.45 were considered to be YB-1/p18 positive (Figure 1), as previously described [12].

### Other tumor markers

Established tumor markers were assessed using the manufacturers’ protocols with reference cut-off values recommended by the manufacturer and validated with internal controls at the Department of Clinical Chemistry and Pathobiochemistry, University Hospital Aachen, Germany. The following assays were used: from Roche, Mannheim, Germany: cancer antigen 125 (CA 125, reference <36 kU/L); carcinoembryonic antigen (CEA, <5 μg/L; CA 15–3, <26 kU/L; CA 19–9, <38 kU/L; CA 72–4, <7 kU/L; cytokeratin fragments 21–1 (CYFRA 21–1, <3.4 μg/L; neuron-specific enolase (NSE), <14 μg/L; alpha-fetoprotein (AFP), <10 μg/L; Dade Behring, Marburg, Germany: beta-2-microglobulin (β2-micro), <1.8 mg/L; Abbott, Wiesbaden, Germany: squamous cell carcinoma antigen (SCC), <1.6 μg/L; DiaSorin S.p.A., Saluggia, Italy: thymidine kinase (TK), <6.8 U/L, tissue polypeptide antigen (TPA), <92 U/L; IBL, Hamburg, Germany: pro-gastrin-releasing peptide (PGRP), <46 ng/L. Due to limited sample availability, these tumor markers were detected in subgroups of the whole cohort.

### Statistics

Results were reported as median and range, and differences between groups were assessed by Mann–Whitney U-test, Kruskal-Wallis-ANOVA, or chi-square-test [14]. The prognostic value of the variables was tested by univariate and multivariate analyses in the Cox regression model. Kaplan Meier curves were plotted to display the impact on survival [15]. The manufacturers’ reference intervals for other tumor markers were used as the cut-offs discriminating tumor marker positivity and negativity, respectively. All statistical analyses were performed using SPSS.

## Results

### Circulating YB-1/p18 is frequently detected in various malignant diseases

**Figure 2:**
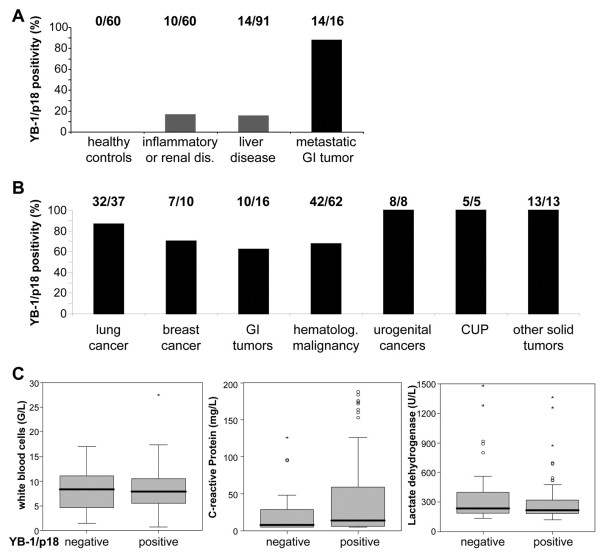
**High prevalence of circulating YB-1/p18 in patients with various malignancies. (A)** YB-1/p18 was previously measured in healthy volunteers (n = 60), patients with inflammatory or renal disease (n = 60), and patients with chronic liver disease (n = 91) with a low rate of YB-1/p18 positivity in patients without malignancies. In contrast, almost all patients with metastatic gastrointestinal tumors (n = 16) tested YB-1/p18 positive. **(B)** YB-1/p18 was detected in plasma samples of patients with malignant diseases with a rate of 62.5% (gastrointestinal [GI] tumors) to 100%. CUP, cancer of unknown primary site. **(C)** Cancer patients with or without detection of plasma YB-1/p18 did not differ with respect to their white-blood cell count (WBC), C-reactive protein (CRP) or lactate dehydrogenase (LDH); P > 0.05, not significant, U-Test. The box-and-whiskers plots display the median, quartiles, range and extreme values. The whiskers extend from the minimum to the maximum value excluding outside (>1.5 times upper/lower quartile, open circle) and “far out” (>3 time upper/lower quartile, asterisks) values.

Our group has recently shown that a YB-1/p18 fragment, detected by immunoblotting with a novel biotinylated monoclonal antibody (Figure 1), can be found in the plasma of patients with malignancies. In a prior study from our group, none of the healthy volunteers (0/60) tested positive for YB-1/p18, whereas 88% of patients with metastatic gastrointestinal tumors (14/16) had detectable plasma YB-1/p18 levels (Figure 2A) [12]. In two cohorts of patients without overt malignancies, but with inflammatory or renal diseases, as well as patients with chronic liver disease, YB-1/p18 positivity was detected in approximately 15% of cases (Figure 2A) [12]. In order to assess the prevalence of YB-1/p18 in malignant diseases, 151 patients (56% male, 44% female, median age 65 years, range 19–84 years) with various malignancies were included in this study (Table 1). The different etiologies of the malignancies are given in Table 1, the stage of remission, tumor staging, and current therapy are listed in Table 2. YB-1/p18 was detected in plasma samples of 77.5% (117/151) of all patients. There was no difference between male (79.8% positive) and female (74.6%) patients (P > 0.05, not significant, Chi-square test, Table 3). Furthermore, the patient’s age had no influence on YB-1/p18 positivity either (not shown).

**Table 1:** Patient characteristics and tumor entities

			**Age [years]**	
	**n**	**%**	**Median**	**Range**
**All patients**	151	100	64.8	18.5- 84.4
Male	84	55.6		
Female	67	44.4		
**Malignancy**				
**Group I: lung cancer**	37	24.5	62.8	43.9- 83.5
Adeno	15	9.9		
Small cell	10	6.6		
Squamous	10	6.6		
Other non small cell	2	1.3		
**Group II: breast cancer**	10	6.6	55.9	34.9- 71.3
Ductal	9	90		
Lobular	1	10		
**Group III: gastrointestinal**	16	10.6	67.5	42.3- 84.4
Stomach cancer	3	2.0		
Colorectal cancer	7	4.6		
Other	6	4.0		
**Group IV: hematological**	62	41.1	64.2	18.5- 83.2
Acute myeloid leukemia	7	4.6		
Chronic myelogenous leukemia	2	1.3		
Hodgkin’s lymphoma	5	3.3		
Non-Hodgkin’s lymphoma	13	8.6		
Other lymphoma	7	4.6		
Multiple myeloma	13	8.6		
Idiopathic thrombocytopenia	5	3.3		
Myelodysplastic syndrome	10	6.6		
**Group V: urogenital cancers**	8	5.3	64.4	38.4- 81.7
Ovarian cancer	2	1.3		
Testicular cancer	2	1.3		
Prostate cancer	1	0.7		
Urinary tract cancer	3	2.0		
**Group VI: CUP**	5	3.3	64.7	41.2- 80.6
**Group VII: other solid tumors**	13	8.6	64.1	30.7- 73.5

**Table 2:** Stage of remission, therapy and metastases at study entry

	**All patients**	**Group I: lung cancer**	**Group II: breast cancer**	**Group III: GI tumors**	**Group IV: hematol.**	**Group V: uro-genital**	**Group VI: CUP**	**Group VII: others**
	**n (%)**	**n (%)**	**n (%)**	**n (%)**	**n (%)**	**n (%)**	**n (%)**	**n (%)**
**Stage of remission**								
ID	99 (65.6)	27 (27.3)	1 (1.1)	9 (9.1)	45 (45.5)	5 (5)	5 (5)	7 (7)
CR	2 (1.3)	0	0	0	2 (100)	0	0	0
PR	4 (2.6)	1 (25)	0	0	2 (50)	1(25)	0	0
NC	2 (1.3)	1 (50)	0	0	1 (50)	0	0	0
PD	44 (29.1)	8 (18.2)	9 (20.5)	7 (15.9)	12 (27.3)	2 (4.5)	0	6 (13.6)
**Metastases**								
CNS	16 (10.6)	8 (50)	0	1 (6.3)	3 (18.7)	2 (12.5)	0	2 (12.5)
Bone	32 (21.2)	9 (45)	6 (18.8)	1 (3.1)	8 (25)	3 (9.4)	2 (6.3)	3 (9.4)
Liver	17 (11.3)	2 (11.8)	2 (11.8)	8 (47.0)	3 (17.6)	2 (11.8)	0	0
Lymphatic	85 (56.3)	21 (24.1)	7 (8)	11 (12.6)	33 (37.9)	5 (5.7)	3 (3.4)	5 (5.7)
**Therapy (at study entry)**								
Chemo	18 (11.9)	4 (22.2)	1 (5.6)	1 (5.6)	4 (22.2)	2 (11.1)	1 (5.6)	5 (27.7)
Radiation	3 (2)	2 (66.7)	0	0	0	0	0	1 (33.3)
Hormone	3 (2)	0	1 (33.3)	0	2 (66.7)	0	0	0

ID, initial diagnosis; CR, complete remission; PR, partial remission; NC, no change; PD, progressive disease.

**Table 3:** Positivity for YB-1/p18 detection in human serum samples of patients with different malignant disorders

	**n**	**YB-1/p18 positivity n/N (%)**
**All patients**	151	117/151 (77.5)
Male	84	67/84 (79.8)
Female	67	50/67 (74.6)
**Malignancy**		
**Group I: lung cancer**	37	32/37 (86.5)
Adeno	15	12/15 (80)
Small cell	10	9/10 (90)
Squamous	10	9/10 (90)
Non small cell	2	2/2 (100)
**Group II: breast cancer**	10	7/10 (70)
Ductal	9	6/9 (66.7)
Lobular	1	1/1 (100)
**Group III: gastrointestinal**	16	10/16 (62.5)
Stomach cancer	3	2/3 (66.7)
Colorectal cancer	7	4/7 (57.1)
Other	6	4/6 (66.7)
**Group IV: hematological**	62	42/62 (67.7)
Acute myeloid leukemia	7	4/7 (57.1)
Chronic myelogenous leukemia	2	1/2 (50)
Hodgkin’s lymphoma	5	4/5 (85.7)
Non-Hodgkin’s lymphoma	13	11/13 (84.6)
Other lymphoma	7	6/7 (85.7)
Multiple myeloma	13	10/13 (77)
Idiopathic thrombocytopenia	5	4/5 (76.9)
Myelodysplastic syndrome	10	2/10 (20)
**Group V: urogenital cancers**	8	8/8 (100)
Ovarian cancer	2	2/2 (100)
Testicular cancer	2	2/2 (100)
Prostate cancer	1	1/1 (100)
Urinary tract cancer	3	3/3 (100)
**Group VI: CUP**	5	5/5 (100)
**Group VII: other solid tumors**	13	13/13 (100)

Among the different etiologies, the vast majority of tested patients with lung cancer (32/37, 86.5%), breast cancer (7/10, 70%), urogenital tumors (8/8, 100%), cancer of unknown primary site (5/5, 100%), and other solid tumors (13/13, 100%) had detectable plasma YB-1/p18 levels (Figure 2B). Patients with gastrointestinal tumors (10/16, 62.5%) and hematological malignancies (42/62, 67.7%) had a lower prevalence of plasma YB-1/p18 levels above the defined threshold. Within the group of patients with hematological malignancies (Table 3), lymphoma (21/25, 84%) or multiple myeloma (10/13, 77%) were more often associated with plasma YB-1/p18 than acute or chronic leukemia (5/9, 56%) or myelodyplastic disorders (2/10, 20%). In contrast, in patients with lung cancer, urogenital carcinomas, or other solid tumors, the histological subtypes did not differ with respect to the YB-1/p18 result (Table 3).

We also analyzed if patients with or without detectable plasma YB-1/p18 levels varied in their laboratory or other clinical characteristics. As shown in Figure 2C, parameters indicating inflammation or (general) tumor load, such as white blood cell count (WBC, median 7.9 G/L in positive vs. 8.3 in negative patients), C-reactive protein (CRP, median 14 versus 8 mg/L), or lactate dehydrogenase (LDH, median 216 versus 234 U/L) did not significantly differ between YB-1/p18 positive and negative patients (P > 0.05, U-test). Furthermore, parameters associated with liver or renal function deterioration did not display significant differences either (data not shown). There was no difference in tumor stage, metastases, or co-morbidities (such as chronic heart failure, arterial hypertension, coronary artery disease, chronic obstructive pulmonary disease, diabetes, renal insufficiency, etc.) between YB-1/p18 seropositive and -negative patients in the total cohort.

### YB-1/p18 is a more sensitive diagnostic biomarker for cancer than established tumor markers

**Figure 3:**
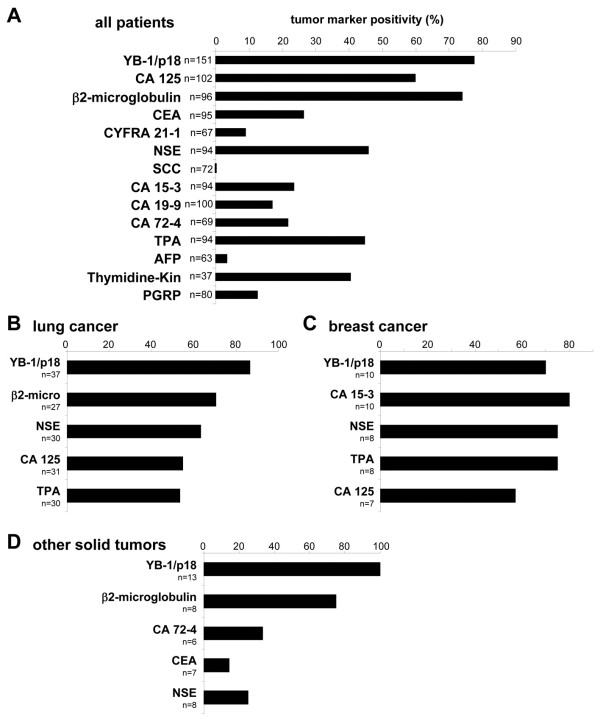
**Comparison of YB-1/p18 with established tumor markers in patients with various malignancies. (A)** Positivity of YB-1/p18 and other available tumor markers for the total cohort of patients with malignant disorders. **(B-D)** Positivity of YB-1/p18 in comparison to the most sensitive of the other tested markers for patients with lung cancer **(B)**, breast cancer **(C)**, or other solid tumors **(D)**. The number of patients in which the respective markers were assessed is given. Abbreviations are used as in the main text. Cut-off values for the established tumor markers are given in the Methods’ section.

A panel of 13 established tumor markers was assessed alongside YB-1/p18 in the cohort of patients with malignancies, namely CA 125, CEA, CA 15–3, CA 19–9, CA 72–4, CYFRA 21–1, NSE, AFP, β2-microglobulin, SCC, thymidine kinase, TPA, and PGRP. YB-1/p18-positivity was not statistically linked to positivity of any of these parameters (cross-table analysis and Chi-square-tests, data not shown). For the total cohort, YB-1/p18 was the marker with the highest sensitivity in detecting malignancies (78% positive, Figure 3A). CA 125 (59%) and β2-microglobulin (74%) also tested positive in the majority of cancer patients, whereas all other markers remained negative in at least half of the patient cohort (Figure 3A).

**Figure 4:**
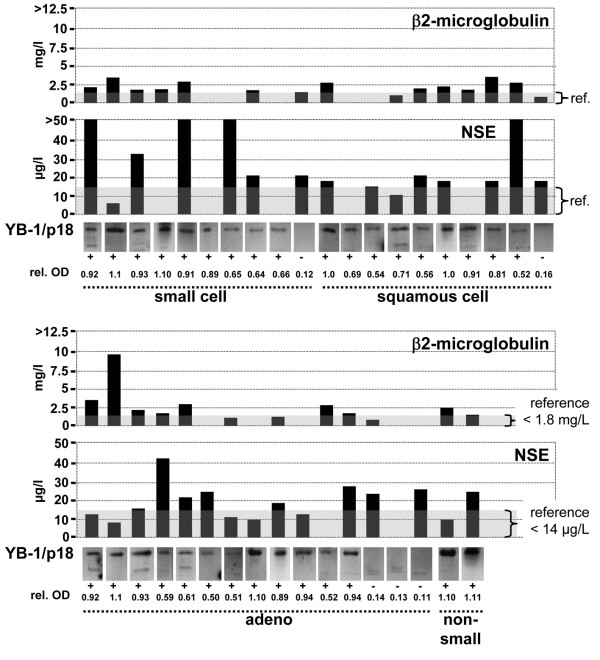
**YB-1/p18, β2-microglobulin, and NSE in lung cancer patients.** Comparison of YB-1/p18 positivity (data from original immunoblotting of serum, relative optical density [rel. OD] of the 18 kDa signal) with individual serum concentrations of β2-microglobulin and NSE (reference interval given in gray). Histological classification of the tumor is given.

When the different tumor entities were analyzed separately, YB-1/p18 positivity was the most frequently detected tumor marker in patients with lung cancer (Figure 3B), urogenital tumors, CUP syndrome, breast cancer (Figure 3C), and the mixed group of patients with other solid tumors (Figure 3D, n = 13) that included very heterogeneous entities such as tumors of the CNS (n = 3), nasopharyngeal tumors (n = 3), or sarcomas (n = 2). Figure 3B and D display the next most sensitive tumor markers for these subgroups, emphasizing that none of the other markers had a similar overall sensitivity comparable to YB-1/p18. In most cases, such as in lung cancer, YB-1/p18 positivity was independent of the histological subtype (Figure 4).

Importantly, some tumor markers had a higher sensitivity than YB-1/p18 in distinct subgroups of patients. For instance, CA 15–3 was more sensitive, while NSE or TPA tested equally sensitive in patients with breast cancer when compared to YB-1/p18 (Figure 3C). In a prior study, we had reported AFP to be more sensitive in detecting hepatocellular carcinoma than YB-1/p18 [12].

### YB-1/p18 detection in plasma varies during therapy, but has limited potential to predict prognosis

**Figure 5:**
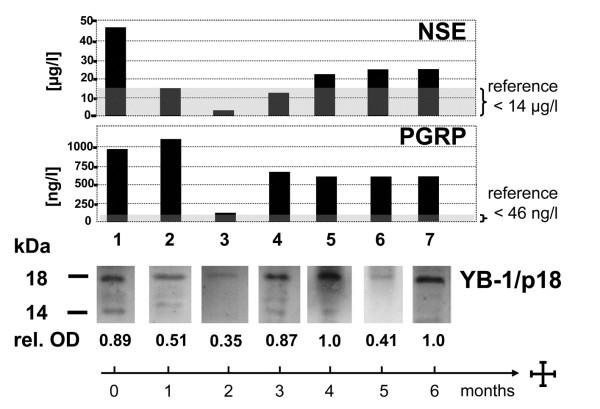
**YB-1/p18 during therapy in a patient with small cell lung cancer.** Serum samples were obtained from a 45-year old male with small cell lung cancer at diagnosis (lane 1) and during chemotherapy with cisplatin/etoposide (lanes 2–3), initially with good response. He was found to progress in month 3 (lane 4), and then was treated with epirubicine, cyclophosphamide, and vincristine (lanes 5–7). Simultaneous measurements of NSE and PGRP are displayed. NSE and PGRP were the only two positive parameters of 13 tumor markers initially tested in this patient. The relative optical density (rel. OD) of the YB-1/p18 signal in immunoblotting is given.

One important clinical value of established tumor markers is their correlation in individual patients with the effectiveness of treatment. In our cohort, we followed 42 out of the 151 patients during the course of therapy. As shown in Figure 5 for an individual patient with small cell lung cancer subsequent to chemotherapy, YB-1/p18 detection in plasma was altered, indicating a response to therapy. Concentrations of other tumor markers that were found positive were also lower after response to therapy. However, truly quantitative assessments of tumor markers were superior to measurement of YB-1/p18 intensity in immunoblotting for predicting individual response to cancer therapy (data not shown).

**Figure 6:**
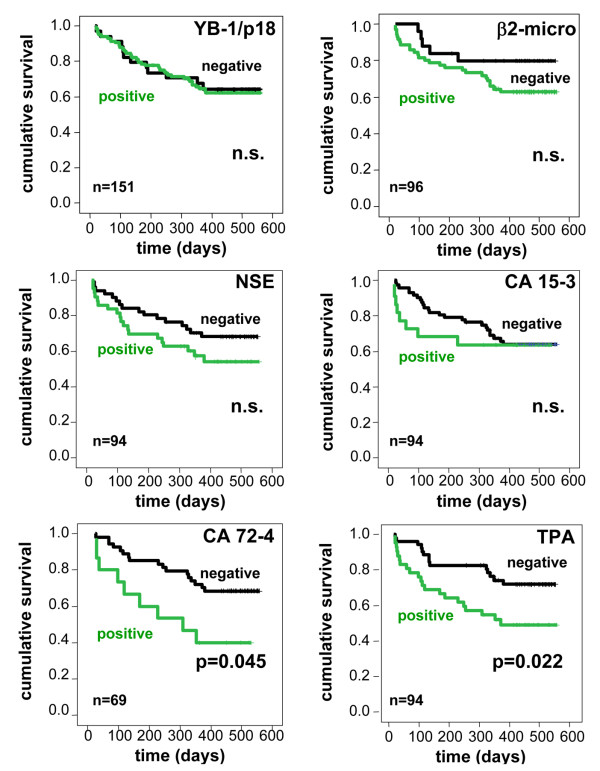
**Prognostic value of YB-1/p18 positivity in comparison to established tumor markers.** Kaplan-Meier curves are depicted to display survival in patients with positive or negative tumor markers, p-values from Cox regression analyses are given; n.s., not significant. The number of patients in which the respective tumor marker has been assessed is given in the figure. Except for CA 72–4 and TPA, that had predictive value for patients’ survival, tumor markers were not related to the prognosis.

Based on the association with histological expression of YB-1 by tumor tissue and unfavorable prognosis (summarized in [2]) we tested whether positivity for YB-1/p18 in serum or other tumor markers were associated with survival. Using Cox regression analysis, YB-1/p18 seropositivity did not predict survival; similar to most other tumor markers (Figure 6). Only positivity for CA 72–4 and TPA was associated with poor prognosis within the cohort of tumor patients, indicating that the tumor markers that were specifically found in subtypes of cancers had a higher probability to have additional prognostic value.

## Discussion

In this study, we set out to test the prevalence and relevance of YB-1/p18 seropositivity in patients diagnosed with different cancerous and leukemic/hematological diseases. Our study cohort was comprised of an unselected heterogeneous group of patients with malignant diseases of various entities and at various stages of disease (e.g., early and advanced, before or during chemotherapy etc.). The main finding of the study is a high positivity rate of the YB-1/p18 in plasma samples of cancer patients. Unexpectedly, this positivity is found irrespective of the underlying cancer provenience and other clinical co-variables that were tested. Such an universal positivity is difficult to understand, given that most cancers derive from specific genetic or epigenetic defects with ensuing alterations of the cellular genome and cancer cell environment [16]. For all the other tested tumor markers, the sensitivity and specificity rates were lower.

The finding of universal seropositivity for circulating YB-1/p18 fragments may be explained by similar widespread YB-1 positivity in tissue samples of cancer patients [2,4,17], emphasizing that YB-1 dysregulation is a common feature found in tumor tissue. So far, immunostaining and analysis of the subcellular distribution of YB-1 from tumor tissue has been used to correlate and predict poor prognosis, especially with nuclear protein accumulation in breast cancer patients [2]. Our findings of circulating YB-1/p18 fragments in plasma of patients with cancer now indicates that dysregulated YB-1 may be prone to be released as fragments into the circulation, which would allow its easy use as a non-invasive disease marker. Similar observations are currently gathered for many micro-RNA (miRNA) in cancerous diseases, as the dysregulation of distinct key miRNA in tumor tissue can be associated with elevated levels of circulating miRNAs in cancer patients [18]. Nevertheless, the profile of circulating miRNAs appears very specific for different types of malignancies or non-malignant diseases [19], while YB-1/p18 detection did not allow us to distinguish between different tumor entities.

It is important to emphasize that YB-1/p18 detection appeared relatively specific for malignant diseases and had a high sensitivity in various malignancies, but its detection, unlike many established tumor markers, was not clearly related to the disease stage or prognosis. The reason for this might be the semi-quantitative nature of our immunoblotting assay. The threshold for YB-1/p18 seropositivity was optimized for sensitivity [12], and immunoblotting quantification against a positive control certainly did not allow linear-range quantification. In fact, in individual patients with several longitudinal YB-1/p18 measurements, there was a moderate association between the quantification of the signal with the response or failure to therapy. Due to the relatively small number of patients with distinct hematological malignancies and the semi-quantitative nature of the immunoblotting method, we were unable to detect a clear association between the number of circulating blasts and the intensity of p18 bands. Thus, a different, more quantitative technique for YB-1/p18 fragment measurement is highly warranted to better estimate the prognostic value of YB-1/p18 in cancerous disease. The development of such an ELISA system, however, is hampered by the fact that currently most antibodies detect not only the p18 fragment, but also full-length YB-1 [13].

It is currently unclear, whether circulating YB-1/p18 fragments are functionally active in patients with malignancies. Inside tumor cells, YB-1 has been shown to fulfill critical cellular functions, such as the transcriptional upregulation of proliferation-associated and downregulation of apoptosis-related genes or induction of drug-transporter genes (like MDR-1) involved in chemoresistance [6,20]. Data from our own laboratories indicated that also extracellular YB-1 may be involved in tumor progression, since adding recombinant YB-1 protein to cancer cell-lines *in vitro* revealed profound pro-mitogenic effects suggesting that secreted YB-1 or its fragments could act as a tumor growth-promoting factor [11]. Further studies are needed to define the exact functions of circulating full-length YB-1 compared to p18 fragments and to define the exact cellular source (tumor vs. stromal cells) of YB-1/p18 in patients with malignant disorders.

Nevertheless, our study demonstrated that circulating YB-1/p18 is highly prevalent in cancer patients and reasonably specific in distinguishing malignant versus non-malignant disorders. One of the challenges in the era of ‘personalized medicine’ is the early detection of cancer, and many biomarkers have failed to be used for screening purposes in clinical practice, even for the most common tumor entities, such as breast or lung cancer [21]. The data provided by our study suggest that it might be highly valuable to incorporate YB-1/p18 measurement into cancer screening approaches. However, it is important to point out that our study comprised a heterogeneous group of patients with different tumor entities and stages. Due to the relatively small patient numbers in the subgroups, our study does not allow to draw specific conclusions for individual tumor entities, e.g. on associations with staging, prognosis or treatment response. Prospective trials with large cohorts are warranted to confirm that circulating YB-1/p18 fragments might be suitable as a general biomarker for the presence of malignant disorders and to assess its potential specific value in distinct tumor entities as a prognostic marker.

## Conclusions

The detection of cold shock proteins, especially of YB-1, by immunostaining in tumor tissue of cancer patients has been related to adverse outcome. Our study now demonstrated that a 18 kD secreted form of YB-1, termed YB-1/p18, carries potential as a circulating biomarker in oncology. By using a novel immunoblotting assay for YB-1/p18 for analyzing YB-1/p18 in plasma of 151 unselected patients with various malignancies, circulating YB-1/p18 had a higher prevalence compared to other established tumor markers and was associated to therapy response in longitudinal assessments. Unlike ‘traditional’ entity-specific cancer biomarkers, YB-1/p18 was largely independent from the histological subtype or stage of disease progression and did not predict the individual prognosis. These data indicate that YB-1/p18 fragments in human plasma may therefore have exceptional potential as a cancer screening marker.

## Abbreviations

AFP: Alpha-fetoprotein; β2-micro: Beta-2-microglobulin; CA 125: Cancer antigen 125; CA19-9: Carbohydrate antigen; CEA: Carcinoembryonic antigen; CRP: C-reactive protein; CSD: Cold shock domain; CYFRA 21–1: Cytokeratin fragments 21–1; CUP: Cancer of unknown primary; Dbp: DNA-binding protein; HMGB: High mobility group box protein; LDH: Lactate dehydrogenase; MDR: Multiple drug resistence; miRNA: micro-RNA; NSE: Neuron-specific enolase; PGRP: Pro-gastrin-releasing peptide; PSA: Prostate-specific antigen; ROC: Receiver operating characteristic; SCC: Squamous cell carcinoma antigen; TGF: Transforming growth factor; TK: Thymidine kinase; TPA: Tissue polypeptide antigen; WBC: White blood cell count; YB-1: Y-box protein-1; YB-1/p18: YB-1 protein fragment p18.

## Competing interests

None of the authors declares competing financial or non-financial interests.

## Authors’ contributions

NK and CSE conducted YB-1 immunoblots. NK, OG and EY performed patient recruitment and provided samples. SB, JAL and UR provided experimental tools and assisted in experiments. FT, NK and PRM designed the study, analyzed data and wrote the manuscript. All authors read and approved the final manuscript.

## Pre-publication history

The pre-publication history for this paper can be accessed here:

http://www.biomedcentral.com/1471-2407/14/33/prepub
